# Balancing selection maintains intraspecific diversity in a deep-sea fish

**DOI:** 10.1038/s41437-025-00813-6

**Published:** 2025-11-27

**Authors:** A. Rus Hoelzel, John Carlos Garza, Anthony Clemento, Georgios A. Gkafas, Natasha Steeds, Michelle Gaither, Harry Peachment, Thomas Regnier, Fiona Gibb

**Affiliations:** 1 Department of Biosciences, South Road, Durham University, Durham, UK; 2 NOAA Southwest Fisheries Science Center and the University of California Santa Cruz, Santa Cruz, CA USA; 3 Department of Ichthyology and Aquatic Environment, University of Thessaly, Volos, Greece; 4 Department of Biology, University of Central Florida, Orlando, FL USA; 5 Marine Directorate, Aberdeen, UK

**Keywords:** Molecular evolution, Genome evolution

## Abstract

Segregating alleles in natural populations can be driven to fixation or loss by genetic drift or directional selection, or may be maintained in a polymorphic state by balancing selection. Balancing selection in a panmictic population is theoretically well established, but not widely understood at the molecular level. In this study, we focus on the evolutionary processes affecting non-synonymous variants at eight functionally relevant loci (based on candidate SNP genotyping) in a deep-sea fish species (*Coryphaenoides rupestris*) that lives across habitat zones ranging from ~200 m to ~2000 m depth. At each of these loci, one allele is predominant in the deeper water. Across a shallower depth range, we find that minor allele frequencies show a highly significant increase or decline progressively across five defined age categories. At single depths below a threshold depth, the deep-water allele declines in frequency with age. Together, these data indicate segregation to different depths, either shallow or deep, and balancing selection to retain variants needed for each depth range. This is supported by signals for long-term balancing selection at these loci (based on published genomic data). We discuss alternative interpretations and conclude that balancing selection maintaining ecotype diversity is the best supported mechanism.

## Introduction

Genetic diversity is the raw material that allows species to adapt to a changing environment (see Hoelzel et al. 2019; Des Roches et al. 2021). Intraspecific differentiation among populations can be maintained by genetic drift and selection when populations are isolated by geographic distance or some environmental feature that restricts gene flow. When populations are parapatric or sympatric and maintain differentiation, there is often post-zygotic isolation (e.g. following allopatric isolation and re-convergence) or some factor that promotes assortative mating (e.g. Class and Dingemanse 2022). The latter can be associated with phenology, as in the classic examples of *Anthoxanthum odoratum* in contaminated or uncontaminated soil (differential flowering times; Antonovics 2006) and the hawthorn fly (*Rhagoletis polmonella*) parasitizing hawthorn berries or apples (different host life cycle; Feder et al. 1988). However, it is also possible for selection to maintain alternative forms, strategies, behaviours and genotypes by balancing or disruptive selection (see van Rijssel et al. 2018; Bitarello et al. 2023). Balancing selection maintains genetic variability in populations by various mechanisms, including frequency dependent selection and heterozygote advantage (Fisher 1923; Charlesworth 2006; Zeng et al. 2021).

For example, polymorphism sustained by balancing selection has been suggested previously for species in the family Centrarchidae (sunfish). The male bluegill sunfish (*Lepomis macrochirus*) can adopt a form that mimics females to achieve stealthy matings and reproductive success, but males adopt this strategy or retain male phenotype, not both strategies, which suggests some type of frequency-dependent selection (Dominey 1980). Both the bluegill sunfish (Ehlinger and Wilson 1988) and the pumpkinseed sunfish (*Lepomis gibbosus*; Parsons and Robinson 2007) also show morphological and behavioural specialisations associated with foraging in different habitats in the same lake. However, we know of no genomic studies for these species that attempt to identify the relevant loci underlying the variation in these traits. The mechanisms are better understood in some lepidopteran systems, for example involving balancing selection at the *cortex* locus controlling wing patterning in butterflies (e.g. Nadeau et al. 2016; Van’t Hof et al. 2016; VanKuren et al. 2019; Wang et al. 2022). In general, while there are various historical examples of balancing selection at the molecular level, such as major histocompatibility loci and sickle cell anaemia (see Hendrick 2007), it hadn’t been thought to be common until recently with the further development of genomics (e.g. Bitarello et al. 2023). At the genomic level, balancing selection affects not just the relevant functional mutation, but also neutral sites in the flanking regions in linkage disequilibrium, maintaining diversity at these sites as well (see Charlesworth 2006). The duration that balancing selection is maintained is often a consequence of the type of selection involved (e.g. frequency dependence may persist longer than heterosis driven by ephemeral pathogen exposure), and this impacts on the footprint that can be detected in the genome indicating balancing selection (Charlesworth 2006).

The deep-sea environment is partitioned with depth by environmental gradients and boundaries associated with factors such as light penetration, hydrostatic pressure, circulation patterns (e.g. Godin et al. 2024) and biological community (known to drive evolutionary change; Gaither et al. 2016). Some species live across a broad depth range, including the roundnose grenadier, *Coryphaenoides rupestris* (Macrouridae), which is found between ~200 and 2000 m in depth (Cohen et al. 1990). Juveniles are thought to be largely pelagic in mid water, and later segregate by depth as adults (Bergstad 1990; Bergstad and Gordon 1994). The species is listed as critically endangered by the IUCN, and Delaval et al. (2018) have found low levels of population genetic structure. Studies on adult *C. rupestris* feeding behaviour show both benthic and pelagic prey, suggesting vertical migration (e.g. Bergstad et al. 2010). Diel vertical migrations (DVM) are common in the oceans, where some species migrate to shallower depths at night to feed and return to deeper water in the day to avoid predation (see Bandara et al. 2021). For example, this behaviour has been documented in the deep sea for the blackspot seabream (*Pagellus bogaraveo*) based on active and passive acoustic telemetry (Afonso et al. 2014). However, a study concluded for *C. rupestris* that ‘although the importance of pelagic prey was found, a diel vertical migration pattern could not be confirmed’ (Høie 2017). It is possible that pelagic prey are taken at the deeper range of the prey’s diel migrations. Both the blackspot seabream (Afonso et al. 2014) and another deep-sea species (*Hexanchus griseus*; Coffey et al. 2020) differ from *C. rupestris* in that they were found only in shallower waters (down to about 700 m). The latter species also showed very consistent habitat depth during each daytime and nighttime period, suggesting that daytime sampling may give consistent results for this and other deep-water species. It is of course also possible that the inference would not extend across species.

A study based on 60 whole genome sequences from *C. rupestris* samples collected along the habitat depth gradient (Gaither et al. 2018) found that individuals captured at depths of 1800 m or greater had fixed genetic differences compared to those from shallower depths at a set of loci associated especially with membranes, morphogenesis and muscle function. Gaither et al. (2018) found that at 750 m to 1500 m depth, genotypes at the identified loci were variable, but all were fixed for a particular allele at 1800 m, implying that the transition was somewhere between 1500 m and 1800 m. The Gaither et al. (2018) study provided the basis for an analysis of those specific loci found to correlate strongly with habitat depth. At least during the daytime periods when samples were collected, the fish in the deepest water habitat appear to remain there based on consistent genotypes. Fidelity to a particular depth range was also suggested from extensive catch per unit effort data (Gaither et al. 2018). Fish captured at different depth also had significant phenotypic differences, consistent with ‘ecotype’ differentiation (Steeds et al. 2024).

Our objective in the current study was to discover the mechanism sustaining ecotype diversity, given that Gaither et al. (2018) showed no differentiation between shallow and deep water ecotypes at neutral genetic markers (and so unlikely to be incipient speciation). To provide the power to test our hypotheses, we increased the sample size per habitat depth (from an average of 15 to an average of 36), the number of depth zones sampled (from 4 to 8) and included age estimates on all 290 newly genotyped fish. Including age estimates allows us to test the hypothesis that the frequency of alleles at loci associated with habitat depth vary with age class, which could be consistent with a pattern promoted by balancing selection retaining multiple phenotypes. If individuals with different genotypes at these loci segregate to depth habitats that suit their genotypes, then any increased mortality risk may only be associated with habitat availability or suitability (which could lead to frequency dependence). An unstable equilibrium (see Prout 1968; Zeng et al. 2021) may be expected if the individuals are mobile among habitats, and not excluded from one habitat or the other, only more successful in the ‘best fit’ habitat. If this was sustained over time, there should be evidence for long-term balancing selection. We use sampling individually aged fish across a broad depth range and genotyping known non-synonymous polymorphisms associated with habitat depth to investigate the potential mechanisms for the retention of ecotype diversity in this species.

## Materials and methods

Specimens of *Coryphaenoides rupestris* were collected from eight depths ranging from 750 m to 1900 m (Table S1). The species is widely distributed along the North Atlantic shelf margins and at the mid-Atlantic Ridge (see Gordon et al. 1992; Priede et al. 2013). Samples were obtained during trawling surveys on the west of the Scotland shelf edge into the Rockall trough over a small geographic range and short period of time (5–14 September 2015). Samples of muscle tissue or fin clips (*N* = 290) were collected as soon after capture as possible and preserved in 20% dimethyl sulfoxide saturated with salt or 95% ethanol before transfer to long-term cold storage at −20 °C. Details of specific sample sets are provided in Table S1. Isolation of DNA was done with a standard phenol/chloroform method. Otoliths (calcified structures from the fish inner ear) were extracted, and the left sagittal otolith was used for age estimation. The otolith was embedded in resin blocks and thinly sectioned transversely through the core for inspection using a binocular microscope. Ages of deep-sea fish are notoriously difficult to determine from the otolith alone, due to long lifespan and periods of slow growth typical of deep-sea fish, so we estimated age by three different methods: (1) independent increment estimates based on otolith ring counts made by three different experienced age readers, (2) pre-anal fin length (PAFL) measurements, given that age estimates showed a strong correlation with the PAFL with a slope close to 1 (Figs. S1, S2) and (3) otolith weights, with the weight range corrected so that it was comparable to the age range categories (Fig. S3). We then took the average to assign the final age estimates. Age estimate variation in comparison with the average is shown in Fig. S4. The average age estimates were further divided into five age classes, 0–5 (*N* = 9), 5–10 (*N* = 13), 10–15 (*N* = 30), 15–20 (*N* = 43), and >20 (*N* = 39) when a subsample from 750 to 1500 m depth was used (see below). To balance sample size per category, the categories were changed to 5–10 (*N* = 13), 10–15 (*N* = 35), 15–19 (*N* = 47), 20–24 (*N* = 48) and >25 (*N* = 13) when the deeper depth ranges (1600 m, 1700–1900 m) were analysed, and >20 used for 1600 m as a fourth category (sample size insufficient for five categories; 5–10: *N* = 7, 10–15: *N* = 19, 15–20: *N* = 10, >20: *N* = 21).

The loci investigated here were chosen based on the study by Gaither et al. (2018), who sequenced 60 genomes of *C. rupestris* across a depth gradient (at 750 m, 1000 m, 1500 m and 1800 m) from the same geographic location and during a short period of time (within a 2 km and over 2 days from a region west of the Hebrides). Genome wide association analysis found strong outlier regions associated with habitat depth including non-synonymous changes in relevant coding loci. We chose 10 SNPs from eight depth-associated loci, and an additional 10 SNPs from three loci that showed no association with depth as controls (Table 1). The eight depth associated loci represented the eight loci showing the strongest signal from the Manhattan plot analysis in the original study, and included five of the loci discussed in detail in Gaither et al. (2018) together with three additional loci. The choice was governed in part by loci for which primers could be designed to amplify reliably and multiplex well. The three control loci were chosen to be out of linkage disequilibrium with the depth-associated loci, and again determined in part by the logistics of the multiplex protocol. Note that loci within a given locus may be affected by linkage disequilibrium, though there is still the potential for differential selection at these sites.

**Table 1:** Statistical tests assessing genotype frequency differences by depth (less than 1700 m compared to 1700 m and deeper) and allele frequencies at 1250 m comparing 14 years or younger to older than 14 years.

Locus	Depth		Age at 1250 m	Depth Allele Freq.	
	*χ*^2^ (*N*)	*p*	Fisher *p* (*N*)	≤14	>14
ROCK1_110	72.3 (245)	<0.00001	0.000074 (35)	0.775	0.133
EGFR1_87	59.7 (274)	<0.00001	0.00014 (40)	0.825	0.275
EEF1D_115	22.2 (245)	0.000015	0.0012 (31)	0.921	0.458
ATG9_60	35.7 (253)	<0.00001	0.0052 (34)	0.85	0.5
OBSL1_58	60.8 (237)	<0.00001	0.000004 (32)	0.816	0.115
b4galt2_84	36.9 (271)	<0.00001	0.00052 (39)	0.8	0.395
adgrl2_36	23.6 (212)	<0.00001	0.011 (29)	0.812	0.385
CAC1E_111	29.7 (216)	<0.00001	0.083 (40)	0.816	0.595
OBSL1_102	27.8 (234)	<0.00001	0.000069 (32)	0.658	0.077
OBSL1_24	49.5 (236)	<0.00001	0.015 (32)	0.842	0.462
**Grid1a_31**	4.18 (224)	0.12	0.45 (32)	-	-
**Grid1a _49**	2.76 (224)	0.25	0.63 (32)	-	-
**Grid1a _52**	0.13 (224)	0.94	0.42 (32)	-	-
**Grid1a _65**	1.09 (223)	0.58	1 (31)	-	-
**Grid1a _100**	0.27 (224)	0.88	0.7 (32)	-	-
**Grid1a _104**	0.3 (224)	0.86	0.5 (32)	-	-
**LRRTM4_25**	1.67 (264)	0.43	0.99 (38)	-	-
**LRRTM4_40**	0.33 (264)	0.84	0.99 (38)	-	-
**LRRTM4_109**	0 (256)	1	1 (38)	-	-
**PKMYT1_61**	0.6 (252)	0.84	0.99 (36)	-	-

For each test (*χ*^2^ or Fisher exact test) the Bonferroni corrected *p* = 0.0025. Loci in bold are the controls. The frequency of the allele most common at depth is shown for the two age groups. *N* = sample size.

To obtain genotype data, sequences from the target loci containing SNP variation associated with depth and the controls were extracted from the 60 genome sequences of Gaither et al. (2018). Consensus sequences were compiled using Sequencher v5.1.1 (Gene Codes Corp.) and target SNP variation identified. Primer 3 v4.1.0 was used with default settings and a target length of 90–143 bp to design primers flanking the target SNPs. The assays were then validated by sequencing the loci using the GT-seq (genotyping in 1000 s by sequencing; Campbell et al. 2015) method, with the modifications described by Baetscher et al. (2018).

Initially, a test sample of 96 individuals were run on a MiSeq (Illumina Inc.) with a 2 × 75 bp paired-end sequencing protocol. This first run was used to assess the relative read-depths among loci, resulting in the dilution of some loci in the GT-seq primer multiplex. We then sequenced these loci in a total of 290 samples using the same protocol. These same 290 individuals were aged from their otoliths. Raw reads were automatically de-multiplexed by the MiSeq (Illumina Inc.) Analysis Software using the individual-specific index barcodes. Paired-end reads were combined using FLASH (min overlap of 4 and max overlap of 50; Magoč and Salzberg 2011). Merged reads were then mapped to the compiled consensus sequences for the target loci using BWA-MEM v0.7.17-r1188 (Li and Durbin 2009). Mapped reads were converted from Sequence Alignment/Map (SAM) files to BAM files with SAMtools v1.13 (Li et al. 2009). Variable sites were identified using FreeBayes v1.3.6 (--haplotype-length 0 -kwVa –no-mnps –no-complex; Garrison and Marth 2012); the positions of all SNPs for each locus were recorded in a VCF file. Raw genotypes were extracted directly from the VCF using vcfR v1.15.0 (Knaus and Grunwald 2017).

Allele frequencies were calculated for each locus in each age and depth category to investigate trends with age for different depths. Significance of changes in allele frequency and tests against Hardy-Weinberg expectations were evaluated using Chi-squared tests, and corrections for false discovery used the Bonferroni method. Trends were assessed by linear regression. Power was high for the Chi square tests using the full dataset assuming an effect size of 0.5 and an alpha of 0.05 (test power = 1.0). For comparisons at a single depth by age using the Fisher exact test (14 or younger: *N* = up to 20, older than 14: *N* = up to 21; see Table 1) the power was greater than 0.70.

To investigate evidence for long-term balancing selection, we used the 60 sequenced genomes (30 below 1500 m and 30 above 1500 m) from Gaither et al. (2018). These sequences provided extended sequence data around the relevant SNPs, but the individuals were not aged and so these samples were only used for the long-term balancing selection analyses. We used three methods, BetaScan2 (Siewert and Voight 2020), Tajima’s D (Tajima 1989) and MLHKA (Maximum Likelihood Hudson-Kreitman-Aguadé test; Wright and Charlesworth 2004). Each of these methods use the statistical analysis of polymorphism data to detect balancing selection. They differ in that BetaScan2 uses both polymorphism and substitution data to detect balancing selection, Tajima’s D compares the number of segregating sites to the average number of nucleotide differences, and MLHKA compares polymorphism within to divergence between species. The VCF file was first filtered to remove sites that were fixed, with option mac set at 1 using VCFtools (Danecek et al. 2011). For BetaScan2, the VCF file was converted to allele count file format and then to betascan format using glactools (Renaud 2018). We calculated beta 1 score for each Contig separately through a sliding window of 1 Kb. Tajima’s D values were calculated in VCFtools (Danecek et al. 2011) also using a 1 Kb window size. Outlier values for Beta 1 were considered those in the upper 5% of Betascan1* scores, while Tajima’s D outliers were those above 95% percentile (after Grace et al. 2021). The significance of the Betascan1* score and Tajima’s D values in that context were assessed using Z-scores. For each position, a Z-score was calculated as Z = μ/σ, where μ is the mean value and σ is the standard deviation. Z-scores with a value greater than 7.72 and 2.42 (two -tailed *p* < 0.05) for Beta scores and Tajima’s D, respectively were considered significant. We also tested for strong Tajima’s D values around the SNP of interest using *t*-test in a 5 Kb window (2.5 Kb before and after the SNP) for both control and depth associated loci.

For the Hudson-Kreitman-Aguadé (HKA) test we used the *C. brevibarbis* genome (Gaither et al. in prep.) for comparisons among species. We used PSMC software (Liu and Hansen 2016) to calculate the time of divergence between *C. rupestris* and *C. brevibarbis*. We trialled different lengths of MCMC chains (100,000–1,000,000) and chose 300,000 (ML values for 100 K: −93.8255, 200 K: −91.2247, 300 K: −88.9939, 500 K: −91.5076, 750 K: −92.5436, 1 M: −93.8255). Segregating sites, pairwise differences, and theta were calculated in DnaSP v.6.0 (Rozas et al. 2017). We set up a model with the depth associated loci under selection and the control loci evolving neutrally. We compared against a model where all loci were considered to be under neutrality. Significance was assessed by likelihood ratio test between the two models using the chi-square statistic (df = number of loci under selection).

## Results

**Fig. 1:**
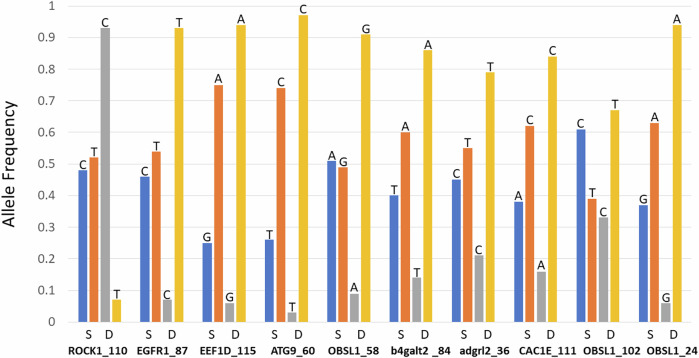
Depth associated allele frequencies. Allele frequencies from 750 to 1600 m (S; shallow) compared to 1700–1900 m (D; deep), showing alleles at each of ten loci showing differentiation with depth. The loci are listed along the x-axis.

We compared the proportion of homozygous genotypes at seven depth categories (combining 750 m and 1000 m) and found an inflection point after 1600 m (average homozygosity change from 750 to 1600 m is 0.527, the average from 1600 to 1900 m is 0.068; Fig. S5). We therefore compared genotype frequencies across two depth zones (750–1600 m and 1700–1900 m) for all loci (Table 1). The division at 1600 m is based on empirical data but is also consistent with theoretical expectations drawn from species distributions in deep water (e.g. many species become intolerant to hydrostatic pressure between 1000 m and 2000 m; see Brown and Thatje 2014). The difference in genotype frequencies between depth zones was highly significant for all of the loci previously identified by Gaither et al. (2018) as associated with depth. In contrast, there were no significant differences between zones at any of the three control loci, confirming their utility for this comparison (Table 1). These differences are illustrated in Fig. 1 for each of the SNPs found to be associated with habitat depth (including three SNPs in OBSL1). Both alleles are segregating for both depth zones at each locus, but one allele clearly dominates in deeper water. The significance of these differences is shown in Table 1. Note that sample size varies somewhat among loci due to differential success with amplification and sequencing. For these larger sample sizes compared to Gaither et al. (2018) the ‘depth’ allele is no longer fixed in the deepest habitats, but it is still clearly the most common allele at depth.

**Fig. 2:**
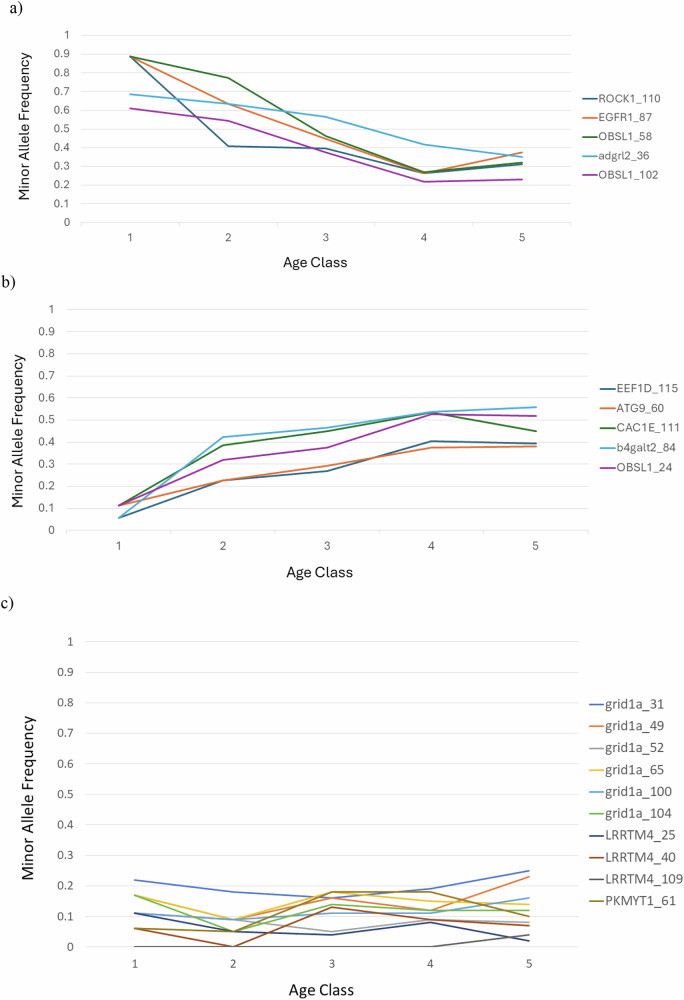
Allele frequencies associated with age. Allele frequency trends for the 750–1500 m range with increasing age class when MAF is **a** declining, **b** increasing, or **c** for the control loci. Keys indicate the different loci.

**Fig. 3:**
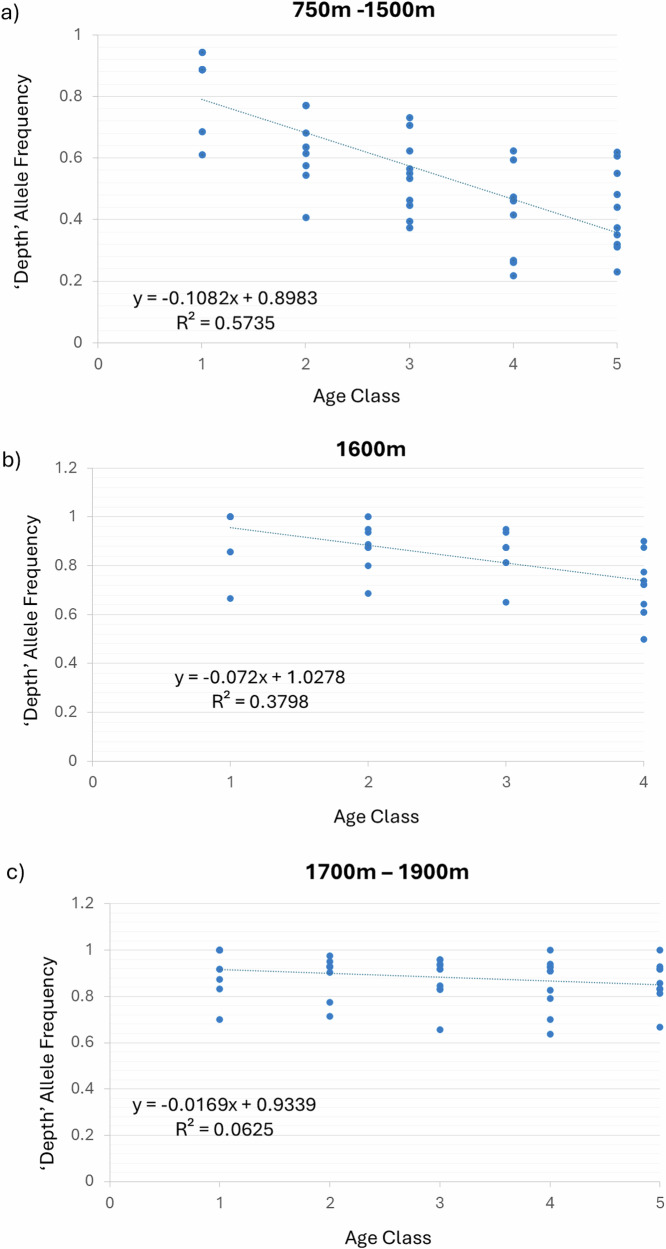
Depth allele frequency trends. Trend for ‘depth’ allele frequency with increasing age class for samples from **a** 750–1500 m, **b** 1600 m only or **c** 1700–1900 m. Regression details are shown in inset.

We then considered the relationship between genotype and age, comparing individuals from the eight depth categories (from 750 to 1900 m) with their estimated age. For our sample set there was no clear linear trend between the age of fish captured and depth (*R*^2^ = 0.053), though the youngest fish (less than age 5) were found only in shallower water (Fig. S6), as reported in Gaither et al. (2018). However, in making comparisons with genotype, we controlled for depth due to the clear association between genotype and depth extremes (Table 1, Fig. 1). When we restricted the depth range to only 1250 m (reflecting a relatively large and broad representation across ages) and compared fish older vs younger than 14 years, all but one depth-associated locus showed significant differentiation (*p* < 0.05) beyond the Bonferroni threshold (see Table 1). The older fish had the lower frequency of the ‘depth’ allele at each locus (allele most common in water deeper than 1600 m), such that genotypes were becoming a better match for the ‘shallow’ water habitat with age (Table 1). We then extended the depth range to 750–1500 m (for sufficient sample size per age class) and tracked the minor allele frequency (MAF) among each of five age classes (Fig. 2). In some cases, MAF tracked downward with increasing age (*R*^2^ = 0.79; *F* = 80.98, *p* < 0.0001; Fig. 2a) and some tracked upward (*R*^2^ = 0.63; *F* = 39.1, *p* < 0.0001; Fig. 2b). None of the control SNPs showed a significant pattern, either at 1250 m (Table 1) or across the five age classes (*R*^2^ = 0.08; *F* = 0.61; *p* = 0.43; Fig. 2c). The lack of significant regression in the controls is not due to a mix of some loci increasing and some decreasing (loci nominally increasing: *F* = 2.47, *p* = 0.127; decreasing: *F* = 1.37, *p* = 0.257). We also assessed frequency variation of the depth allele across the five age classes for fish at 750–1500 m, 1600 m and for the range 1700–1900 m (Fig. 3). At 750–1500 m (*F* = 64.5, *p* < 0.0001; Fig. 3a) and 1600 m (*F* = 23.27; *p* < 0.0001; Fig. 3b), the depth allele decreased with increasing age class for all 10 SNPs that had been associated with habitat depth variation. In the deepest water (1700–1900 m), the depth allele frequency was high and relatively stable across all ages (*F* = 3.2; *p* = 0.08; Fig. 3c).

We tested for potential deviations from the Hardy-Weinberg equilibrium (HWE) either side of the putative depth threshold separating the two groups (750–1600 m compared to 1700–1900 m). There were no significant deviations from HWE expectations within these depth ranges for any loci (Table S2). However, when all samples were included there were significant deviations (heterozygote deficiency) for four of the loci. This was most likely associate with a Wahlund effect, due to the strong allele frequency difference between the two depth ranges at those loci. In Gaither et al. (2018), loci showing an association with depth based on Manhattan plot analyses demonstrated elevated linkage disequilibrium (*R*^2^) compared to neutral loci, suggesting that they evolve as a haplotype (Gaither et al. 2018). The strongest linkage disequilibrium signal was for the non-synonymous SNPs used in this study. Here we assessed the frequency at which for a given locus, a genotype fixed at one allele for either the depth or the alternative allele, was also fixed the same way at other loci. This happened 82.4% of the time, and was significantly more frequent than expected by chance (*χ*^2^ = 1088, *p* < 0.00001), consistent with the loci evolving together.

**Fig. 4:**
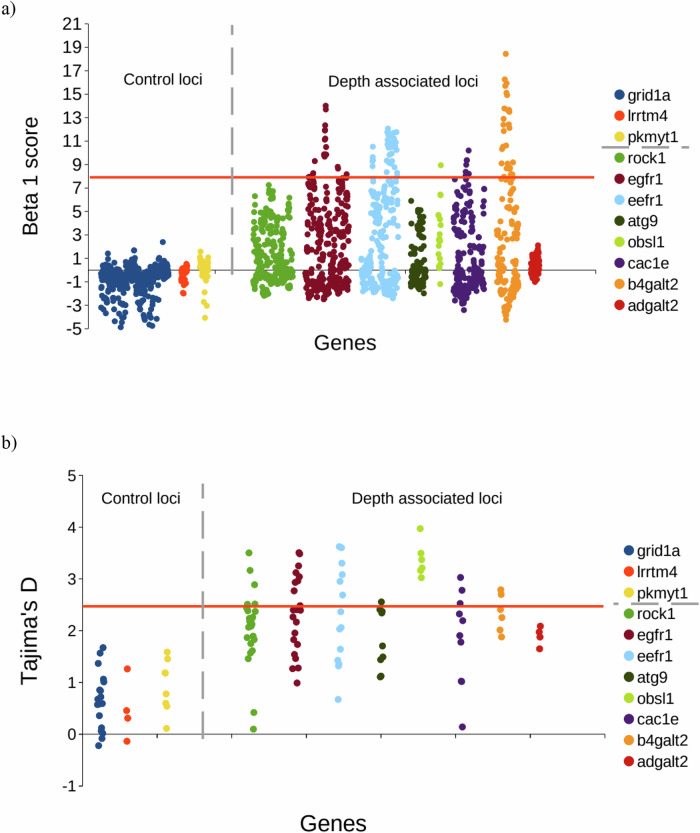
Evidence for balancing selection. **a** Beta 1 scores in sliding 1Kb window. Orange line indicate threshold of top 5% of beta scores (7.74). **b** Tajima’s *D* values based on 1 Kb window. Orange line indicates threshold of top 95% precentile (2.4). The *x*-axis shows the position along the contig relevant to each locus. Fewer dots in part (**b**) are due to sensitivity of the analysis to missing data. Data used for analysis are from Gaither et al. (2018). The vertical grey dashed line separates control from test loci.

Evidence for long-term balancing selection is presented in Table 2 and Fig. 4. Beta 1 scores ranged from −5.22 to 18.44. The top 5% of scores were above 7.74. All control loci had score values well below the threshold, while all loci associated with depth were elevated or above 7.74, with the exception of adgalt2 (Fig. 4). Tajima’s D showed a similar pattern for the 1 Kb window analysis (Fig. 4). Tajima’s D values around the SNP of interest showed significance for all depth associated loci, but none of the control loci (Table 2). For MLHKA the likelihood value for the neutral model was −107,525 and −88.9939 for a model of 8 loci under selection. To test for significance, we used the likelihood ratio test (twice the difference in log likelihood between the models) and the chi-squared distribution. The ratio was significant (*χ*^2^ = 37.06, *p* < 0.001, *df* = 8), indicating that the eight depth associated loci fit a balancing selection model better than the neutral model.

**Table 2:**
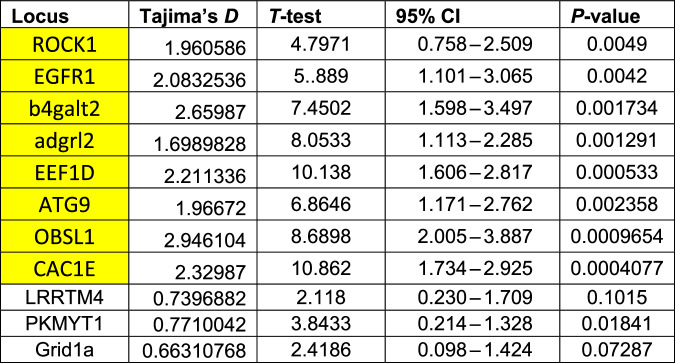
Results for Tajima’s *D* test using 5 Kb window around relevant SNP (data from Gaither et al. 2018). Loci associated with depth highlighted in yellow.

## Discussion

The non-synonymous variants identified in Gaither et al. (2018) again showed a strong correlation between habitat depth and allele frequency in the deep-sea fish, *C. rupestris*. All of the loci identified earlier showed a highly significant pattern, while none of the control loci did (Table 1). Based on our larger sample size and broader sampling range, we found that the transition between selection regimes seems to occur between 1600 m and 1700 m depth (Fig. S5), and while one allele is most common in deeper water (1700–1900 m), both alleles were present throughout the depth range. The loci that showed a strong association with depth also showed allele frequency variation correlated with age when controlling for depth (Figs. 2, 3 and Table 1). None of the control loci showed either pattern or association. There was no clear correlation between age and depth for adult fish (Fig. S6), instead a broad age range was found at all depths.

The loci that correlate with both depth and age are associated with membranes, development and muscle contraction. All of these functions are potentially associated with adaptive needs at different depths. For example, ROCK1 is understood to promote the formation of migrosomes (vesicles generated during cell migration), which are essential for embryonic organ development during morphogenesis in zebrafish (Jiang et al. 2019). Steeds et al. (2024) show that *C. rupestris* and three other species with similar depth distributions have morphological variation associated with different depths (especially associated with gape size, maximum width, swim bladder weight, and body elongation in *C. rupestris*). This is consistent with a large proportion of the loci putatively under selection associated with habitat depth in *C. rupestris* being involved in development or morphogenesis (Gaither et al. 2018). ROCK1 is also within a genomic region correlated with adult migration timing to freshwater in Pacific salmon species (Thompson et al. 2020; Willis et al. 2020). However, this association is shared with the neighbouring locus, GREB1L, an oestrogen-responsive gene, and structural variation in the intergenic region between them. It is not yet clear what role ROCK1 plays in migration timing, if any (as opposed to the association being due to linkage disequilibrium with the intergenic region or GREB1L). EGRF1 also plays an important role in development and has been implicated in the growth of tumours (Fromm et al. 2008). EGRF1 showed a particularly strong association with depth (Fig. 1). Other depth-associated loci are involved in membrane function (Table S3), including CAC1E which provides voltage regulated calcium channels involved in functions such as muscle contraction. OBSL1 also plays a role in muscle contraction (Geisler et al. 2007). Information on the function of all eight loci is provided in Table S3.

Figure S6 shows that there is no clear association between habitat depth and fish age, except for a lack of fish younger than 5 years in water deeper than 1250 m. Fish older than 5 years were found across all depths (Fig. S6). Although individual fish segregate to a particular depth as adults, the specific depth they segregate to is not associated with their age. This means that depth segregation by age can’t fully explain the progressive increase or decrease in MAF, especially when measured at a restricted depth or depth range. The broadest age range was found at 1250 m (Table 2, Fig. S6), which permitted a comparison between young and old fish at a single depth, showing significant differentiation between young and old fish at that depth (Table 1). When the depth range was restricted to 750–1500 m, there was a consistent decrease or increase in MAF (which could be either the depth or shallow related allele) with increasing age class for all depth-related loci (Fig. 2). This could happen in an unstable equilibrium if both ecotypes benefit from segregating to an appropriate habitat depth, but the capacity of each habitat varies over time or individuals don’t show strict fidelity to a given depth. The fact that the frequency of the depth allele decreased with age at all depths below a threshold depth of 1700 m could be interpreted several ways. It could suggest that fish with the depth allele migrate to deeper water increasingly as they age. However, in that case we would expect to see the depth allele increasing with age in the deeper water. Instead, there is a non-significant trend for it to decrease with age there, and all ages show high frequency for the depth allele at 1700–1900 m. It is possible that further sampling in deeper water would show the depth allele increasing, though it is more likely that the alleles are near or at their upper limit (fixation) at that depth. Alternatively, older fish in shallower water may die younger if they have the depth allele. This may be unexpected in a highly mobile species such as *C. rupestris* that can adjust to different habitat depths; however, a system involving antagonistic pleiotropy is possible. Under this scenario the depth allele would be beneficial or neutral in shallower water when the fish is young, and detrimental in shallower water when they are old. It would seem to be beneficial at all ages in the deepest water.

Allele frequency could also change with age in a sample if there is a sampling bias, which may be associated with different ages being sampled at different depths, or with diel patterns of movement. Neither are supported by the data for this study. DVM could affect the observed pattern (if there was movement during the day) but that would be more likely to disrupt rather than create the clear associations between genotype, age and depth. Further, there was a significant correlation between allele frequency and age class when three different controls for depth were applied (Table 1, Figs. 2, 3), and fish at a given depth were sampled at various times of day. Another potentially relevant factor is that the proportion of large fish (and therefore old fish) was seen to increase as a result of decreased fishing pressure in the Rockall Trough, particularly in the shallower depths (Mindel et al. 2018). Since allele frequencies at these loci varied with age, this raises the possibility that fishing could have impacted allele frequencies. Although close linkage (SNPs within the same locus) may be expected to show correlated patterns of change with age, this was not always the case (see Fig. 2), which could be due to either drift or selection.

Although our measurements of the pattern of allele frequency across habitat depths may have been distorted by these factors, the fact that two alleles are retained in the breeding population at each of these loci suggests that some type of selection is promoting their retention. A progressive change in allele frequency with age in this long-lived species may suggest balancing selection by frequency dependence. In a recent study based on the very large Biobank sample of human genomes (276,406 participants from the UK), Long and Zhang (2023) tested the hypothesis that pleiotropic mutations that promote reproduction but cause aging in humans are favoured by natural selection. They compared age cohorts born between 1940 and 1965 and found alleles at relevant loci with the expected antagonistic pleiotropy characteristics (improved reproduction but shorter lifespan) that showed a positive association with cohort age. For example, the T allele at chromosome position 6p25.3, associated with expression at IRF4, was ‘associated with a younger age at first sexual intercourse and an increased risk of mortality and shows a rise in the frequency of the T allele over 25 years’ (Long and Zhang 2023).

Our data suggest balancing selection retaining diversity, but probably not by overdominance, which might be expected to lead to an equilibrium with stable allele frequencies. The observed dynamic allele frequencies, trending both up and down, also seems incompatible with underdominance (disruptive selection). The data instead seem most consistent with balancing selection and an unstable equilibrium, based on occupation of heterogeneous habitat and the differential segregation of individuals with different genotypes. The potential for free movement across habitats together with improved reproduction in the best suited habitat, could explain the dynamics. Each allele could be favoured in the right environment, and so the ‘depth’ allele may increase or decrease, depending the relative frequency of ecotypes and habitat availability. If individuals were instead restricted to a specific depth range, then the allele frequency changes (see Figs. 2 and 3) would suggest early mortality for genotypes in the ‘wrong’ habitat, and the degree of change may suggest a strong effect. However, our precision with detecting allele frequency may be impacted by noise generated from individual movements among habitats and the chance composition of catches at a given depth. Therefore, trending up or down may be more informative that the exact allele frequencies. The fact that allele frequencies trend up or down is most consistent with balancing selection associated with segregation among habitats, and differential reproductive success rather than high mortality. There is some indication that *C. rupestris* is a regional and seasonal spawner (Bergstad 1990), and potential breeding swarms have been reported at 1500 m (Neat 2017). If fish from different depths gather together to mate, this would be consistent with the lack of differentiation found at neutral loci (Gaither et al. 2018), and the persistence of polymorphisms in a single panmictic population.

We find that for loci associated with deep-water habitat, MAF changes across age classes, and that this holds even when examining fish collected from a single or restricted habitat depth (Table 1, Fig. 2). The persistence of variation at these loci is likely related to variation in their spatial and temporal frequencies, and due to balancing selection retaining both alleles. These loci also showed evidence of long-term balancing selection based on polymorphism data (Table 2, Fig. 4), and none of the control loci showed evidence for this. For a single species to exploit habitat across a range where habitat characteristics (associated with light, pressure, prey resources, etc.) vary extensively, it is likely necessary to maintain polymorphism that allows individuals to be best adapted to one or another of the different depth habitats. This could be associated with life stage (e.g. differential segregation for juveniles and adults), but there is little evidence for that in this case (see Fig. S6). Eventually this may lead to segregation by habitat and assortative mating, but there is no evidence that this has happened yet in this species (*Fst* values for 44,650 neutral loci comparing samples from different habitat depths were not significantly different from zero; see Gaither et al. 2018). Instead, it seems that the polymorphism is maintained within a reproductive population. Bolnick et al. (2003) review many examples of conspecific individual specialisation and the potential evolutionary mechanisms, including directional, balancing and disruptive selection. Bitarello et al. (2023) provide a timeline from the first mention of balancing selection by Fisher (1923) to modern studies using genomic data to detect signals of balancing selection. Here we identify some of the molecular mechanisms that appear to underlie the polymorphism supporting individual differences associated with divergent habitat in the deep sea. Of the various potential mechanisms, the data suggest frequency dependent balancing selection as the most likely mechanism, impacting loci with functions relevant to development and morphology.

## Supplementary information

Supplement

## Data Availability

Genotype data for this study can be found at Dryad (10.5061/dryad.34tmpg4xx).
