# Supplementary material for: Balancing selection maintains intraspecific diversity in a deep-sea fish
Source: Heredity (Edinb). 2025 Nov 27;135(1):13–22. doi: 10.1038/s41437-025-00813-6 (PMC12811240; doi:10.1038/s41437-025-00813-6)

**Balancing selection maintains intraspecific diversity in a deep-sea fish**

A. Rus Hoelzel, John Carlos Garza, Anthony Clemento, Georgios A. Gkafas, Natasha Steeds, Michelle Gaither, Harry Peachment, Thomas Regnier and Fiona Gibb

**Supplement**

| **Range** |  | **ROCK1_110** | **EGFR1_87** | **EEF1D_115** | **ATG9_60** | **OBSL1_58** | **CAC1E_111** | **b4galt2_84** | **adgrl2_36** |
| --- | --- | --- | --- | --- | --- | --- | --- | --- | --- |
| 750-1250 | χ^2^ | 6.47 | 6.74 | 3.06 | 0 | 4.32 | 0.08 | 0.05 | 0.63 |
|  | p | 0.039 | 0.034 | 0.21 | 1 | 0.11 | 0.96 | 0.97 | 0.72 |
| 1700-1900 | χ^2^ | 0.72 | 0.03 | 0.06 | 0 | 0.69 | 1.29 | 0.41 | 2.32 |
|  | p | 0.7 | 0.86 | 0.97 | 1 | 0.71 | 0.52 | 0.81 | 0.31 |
| All | χ^2^ | **29.35** | **24.9** | **11.9** | 3.78 | **27.9** | 0.04 | 0.36 | 0.13 |
|  | p | **<0.00001** | **<0.00001** | **0.002** | 0.15 | **<0.00001** | 0.98 | 0.83 | 0.93 |

Table S1: Tests for compliance with the Hardy Weinberg Equilibrium at different depth ranges. Test results shown in bold are significant past the Bonferroni correction.

Table S2: Putative gene functions (from UniProt and Gene Card). Loci in italics are controls.

| **Locus** | **Functions** |
| --- | --- |
| ROCK1 | Involved in regulation of smooth **muscle contraction**, actin cytoskeleton organization, stress fiber and focal adhesion formation, neurite retraction, cell adhesion and motility via phosphorylation. Also thought to be involved in fish migration. |
| EGFR1 | **Transmembrane** protein that is a receptor for members of the epidermal growth factor family (EGF family) of extracellular protein ligands. |
| EEF1D | Part of the EEF1 protein complex, translation elongation factors involved in **development** and overexpressed in tumors. |
| ATG9 | Cycles between the preautophagosomal structure/phagophore assembly site (PAS) and the cytoplasmic vesicle pool and **supplies membrane** for the growing autophagosome. |
| OBSL1 | Cytoskeletal adaptor proteins function in linking the internal cytoskeleton of cells to the **cell membrane**. This gene encodes a cytoskeletal adaptor protein, which is a member of the Unc-89/obscurin family. Also involved in structural regulation of the M-band in muscles. |
| b4galt2 | **membrane-bound** glycoproteins that appear to have exclusive specificity for the donor substrate UDP-galactose |
| adgrl2 | Member of the latrophilin subfamily of G-protein coupled receptors. The proprotein is thought to be further cleaved within a cysteine-rich G-protein-coupled receptor proteolysis site into two chains that are non-covalently bound at the **cell membrane**. |
| CAC1E | Involved in a variety of calcium-dependent processes, including **muscle contraction**, hormone or neurotransmitter release, gene expression, cell motility, cell division and cell death. |
| *Grid1a* | Receptor for glutamate that functions as a ligand-gated ion channel in the central nervous system and plays an important role in excitatory synaptic transmission. |
| *LRRTM4* | May play a role in the development and maintenance of the vertebrate nervous system. Exhibits strong synaptogenic activity, restricted to excitatory presynaptic differentiation (By similarity). |
| *PKMYT1* | Acts as a negative regulator of entry into mitosis (G2 to M transition) by phosphorylation of the CDK1 kinase specifically when CDK1 is complexed to cyclins. |

Table S2: Sampling for genotyping analysis (for 1000m, parenthetical range combines 750m and 1000m).

| **Sampling Depth (m)** | **Number** | **Age Range** | **Mean Age & s.d.** |
| --- | --- | --- | --- |
| 750 | 6 | 2.8 – 17 | 9.6 ± 5.36 |
| 1000 | 50 | 8 – 25.6 | 16.35 ± 4.35 (15.63 ± 4.89) |
| 1250 | 41 | 1 – 29.4 | 14.52 ± 8.53 |
| 1500 | 37 | 9.4 – 27.2 | 18.2 ± 4.47 |
| 1600 | 50 | 5.2 – 28 | 17.49 ± 6.45 |
| 1700 | 38 | 9.2 – 25.2 | 17.07 ± 4.18 |
| 1800 | 37 | 8 – 27.6 | 19.11 ±4.99 |
| 1900 | 31 | 7.4 – 29.2 | 18.73 ± 5.19 |

Figure S1: Proportion of genotypes homozygous for the ‘depth’ allele in relation to habitat depth.


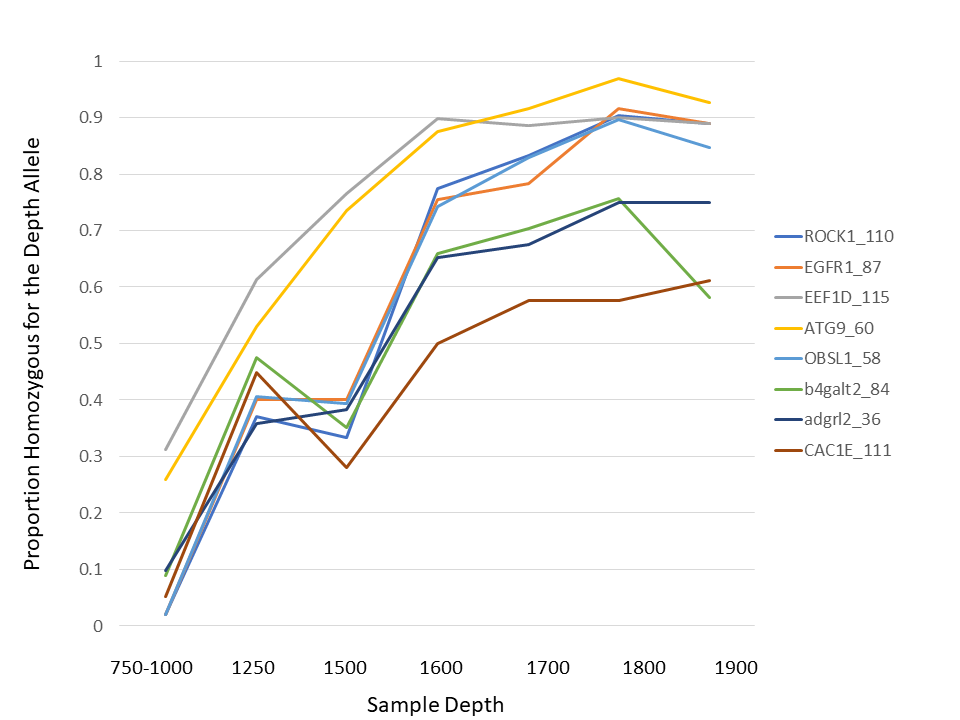


Figure S2: Relationship between sample depth and average age estimate.


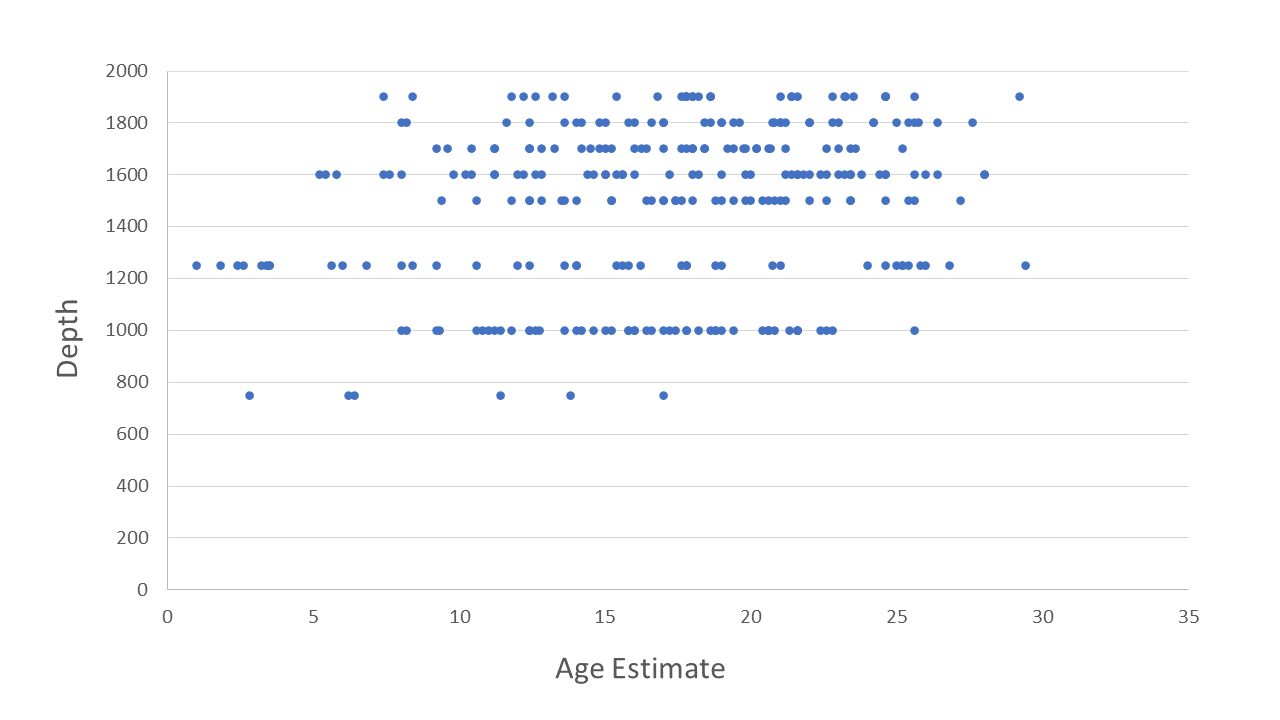


Figure S3: Regression between Pre-anal fin length (PFAL) and the first age estimate from otoliths.


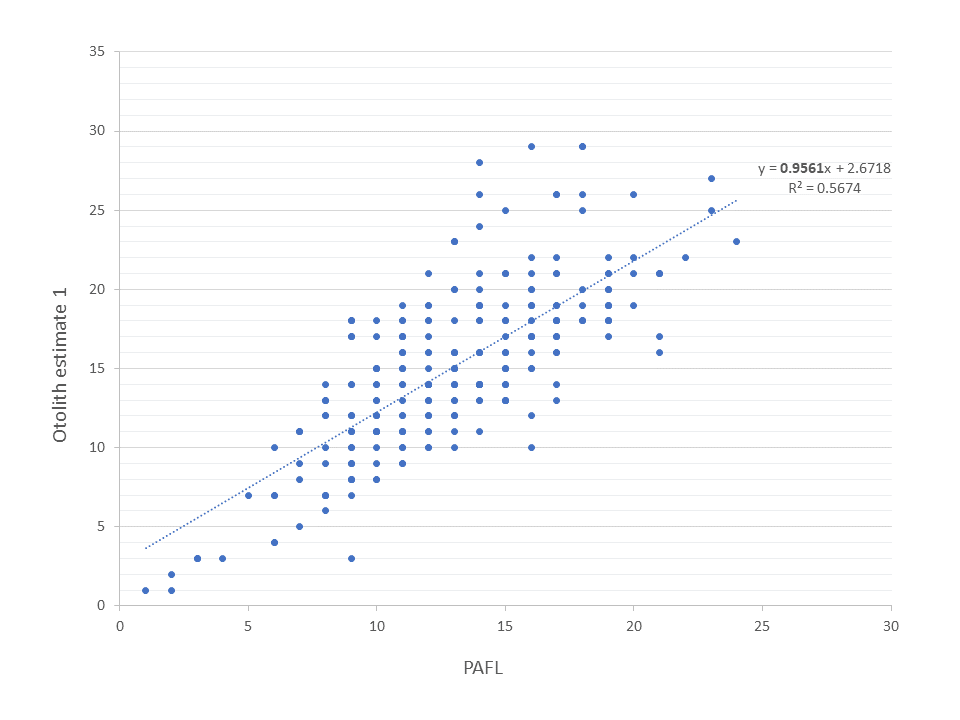


Figure S4: Regression between Pre-anal fin length (PFAL) and the two further age estimates from otoliths.


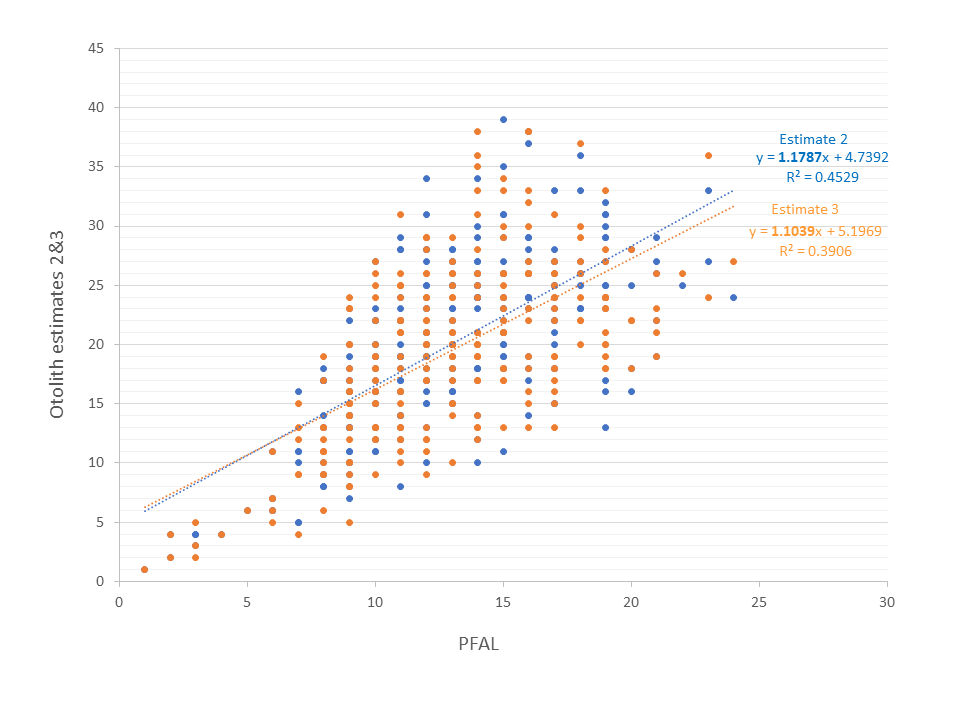


Figure S5: Correction factor between otolith weight (mg) and otolith ‘class’ used as the age metric.


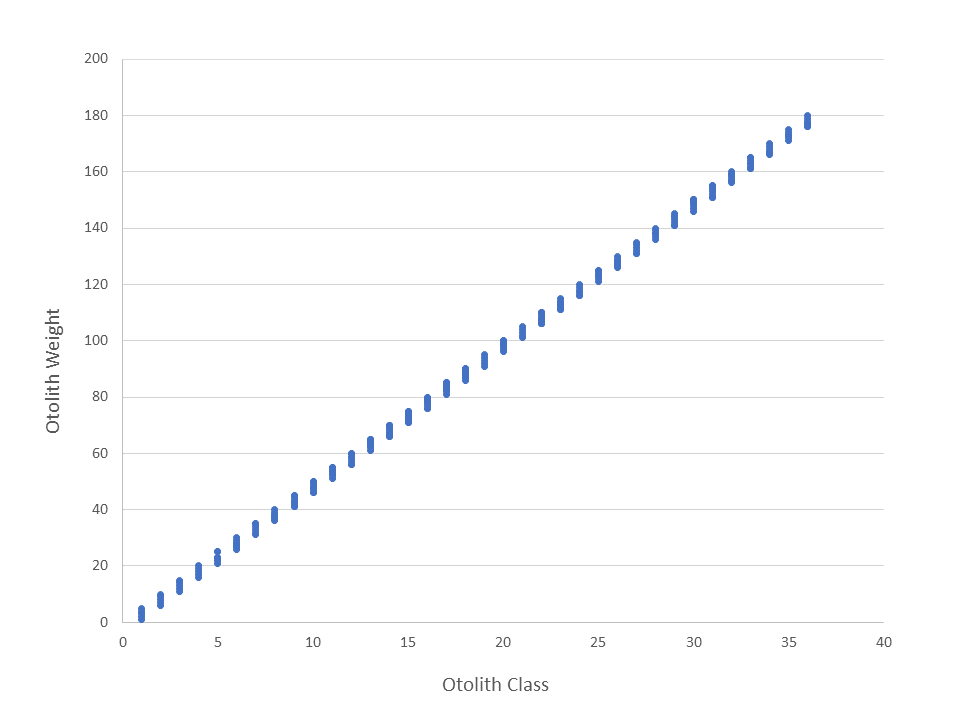


Figure S6: Relationship between the average and each composite age estimate.


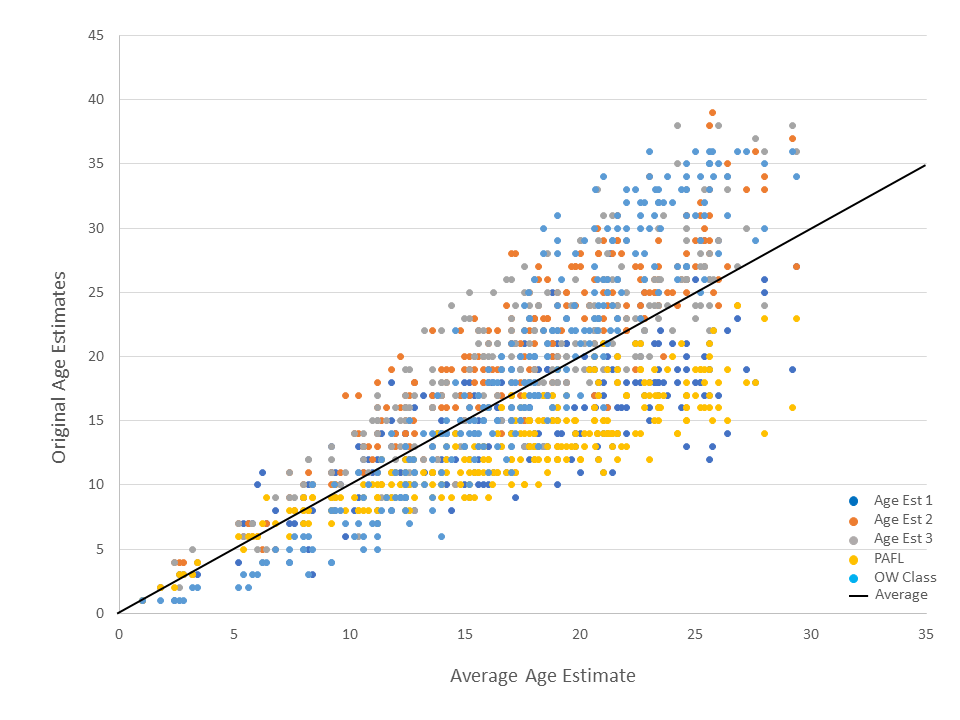

Supplement: Supplementary file 1 — Supplement [file 41437_2025_813_MOESM1_ESM.docx]
